# Community-based ART distribution system can effectively facilitate long-term program retention and low-rates of death and virologic failure in rural Uganda

**DOI:** 10.1186/s12981-015-0077-4

**Published:** 2015-11-12

**Authors:** Stephen Okoboi, Erin Ding, Steven Persuad, Jonathan Wangisi, Josephine Birungi, Susan Shurgold, Darius Kato, Maureen Nyonyintono, Aggrey Egessa, Celestin Bakanda, Paula Munderi, Pontiano Kaleebu, David M. Moore

**Affiliations:** Mulago Hospital Complex, The AIDS Support Organization (TASO), P.O BOX 10443, Kampala, Uganda; The BC Centre for Excellence in HIV/AIDS, Vancouver, Canada; Faculty of Medicine, University of British Columbia, Vancouver, Canada; MRC/UVRI Uganda Research Unit on AIDS, Entebbe, Uganda

**Keywords:** Antiretroviral therapy, Virologic failure, Lost-to-follow-up; mortality, Retention, Sub-Saharan Africa, Uganda

## Abstract

**Background:**

Community-drug distribution point is a care model for stable patients in the community designed to make ART delivery more efficient for the health system and provide appropriate support to encourage long-term retention of patients. We examined program retention among ART program participants in rural Uganda, which has used a community-based distribution model of ART delivery since 2004.

**Methods:**

We analyzed data of all patients >18 years who initiated ART in Jinja, Ugandan site of The AIDS Support Organization between January 1, 2004 and July 31, 2009. Participants attended clinic or outreach visits every 2–3 months and had CD4 cell counts measured every 6 months. Retention to care was defined as any patient with at least one visit in the 6 months before June 1, 2013. We then identified participants with at least one visit in the 6 months before June 1, 2013 and examined associations with mortality and lost-to-follow-up (LTFU). Participants with >4 years of follow up during August, 2012 to May, 2013 had viral load conducted, since no routine viral load testing was available.

**Results:**

A total of 3345 participants began ART during 2004–2009. The median time on ART in June 2013 was 5.69 years. A total of 1335 (40 %) were residents of Jinja district and 2005 (60 %) resided in outlying districts. Of these, 2322 (69 %) were retained in care, 577 (17 %) died, 161 (5 %) transferred out and 285 (9 %) were LTFU. Factors associated with mortality or LTFU included male gender, [Adjusted Hazard Ratio (AHR) = 1.56; 95 % CI 1.28–1.9], CD4 cell count <50 cells/μL (AHR = 4.09; 95 % CI 3.13–5.36) or 50–199 cells/μL (AHR = 1.86; 95 % CI 1.46–2.37); ART initiation and WHO stages 3 (AHR = 1.35; 95 % CI 1.1–1.66) or 4 (AHR = 1.74; 95 % CI 1.23–2.45). Residence outside of Jinja district was not associated with mortality/LTFU (p value = 0.562). Of 870 participants who had VL tests, 756 (87 %) had VLs <50 copies/mL.

**Conclusion:**

Community-based ART distribution systems can effectively mitigate the barriers to program retention and result in good rates of virologic suppression.

## Background

Large-scale antiretroviral treatment (ART) programs for HIV and AIDS were launched in sub-Saharan Africa in 2003–2004 with the increases in funding made available through the Global Fund Against AIDS, Tuberculosis and Malaria and the President’s Emergency Plan for AIDS Relief (PEPFAR) [1–3]. From their inception, much attention was then focused on patients’ day-to-day adherence to ART which have generally shown very good results [1, 3–5].

However, long-term retention of patients in treatment programs, a prerequisite for achieving any adherence at all has gained more attention in recent years [4]. Most large-scale treatment providers have few resources available to track missing patients; and patient attrition is often not prioritized as a primary outcome and clinical studies often focus solely on describing those patients who are retained in care. In a study conducted in 17 facilities in Uganda, Tanzania and Zambia, loss to follow up ranged from an average of 25.9 % at 1 year to 41.9 % at 4 years [8]. Other studies have shown that transportation costs, absence of treatment partner or lack of family support as significant barrier to program retention [6, 7, 12, 16, 24].

In studies with follow-up beyond the first 4 years of treatment, LTFU has been shown to vary between 7 % in a program in Malawi [6, 10] to 56 % for one program in Uganda [7]. In a recent systematic review of participant retention in ART programs in Africa, researchers found an estimated median retention of care of 64.4 % (range 57.5–72.1 %) after 3 years after ART initiation among 39 cohorts with over 225,000 patients in total [8]. LTFU was the most common factor for not been retained [13, 18]. Possible explanations for these variations in patient retention include patients having to pay for their medication, patients accessing care at another facility and unreported mortality [6, 10 16]. Additionally, lack of transportation and distance from the clinic, financial strain, work or child care responsibilities, social issues, and feeling that their health was too poor or too good to warrant continuing have all been identified as barriers to being retained in care [6, 10, 16]. When investigated, the proportion of patients LTFU who were then discovered to have died ranged from 25 to 50 % [1, 10, 17, 19].

We undertook an analysis of program data at one of the TASO centers in Jinja, Uganda to examine program retention, LTFU and mortality among clients enrolled in the first 5 years of the ART program. Our primary interest was in examining whether clients from districts which were distant from the TASO centre had the same likelihood of being retained in care, as those who resided close to the TASO clinic. In a sample of those retained in care after 5 years of treatment, we also conducted VL testing to measure the prevalence of virologically defined treatment failure in a program without access to routine VL testing.

## Results

A total of 3340 participants began ART during 2004–2009 and the median time on ART for these individuals in June 2013 was 5.7 years (IQR = 4.1–7.2). Of these individuals, 2379 (71 %) were females, the median age at ART initiation was 40 years (IQR = 34–46), and 1618 (55 %) of the participants had some primary education as their highest level attained. The median CD4 cell count at initiation was 184 cells/μL (IQR = 95–298). A total of 1335 (40 %) were residents of Jinja district and 2005 (60 %) resided in outlying districts. A total of, 1361 (44 %) of the participants began treatment on Nevirapine, Zidovudine and Lamivudine, 950 (31 %) began with Niverapine, Stavudine and Lamivudine and 323 (11 %) began with Efavirenz, Zidovudine and Lamivudine.

A total of 2317 (69 %) participants had at least one recorded clinic or outreach visit recorded between November 1, 2012–May 31, 2013. For participants not retained in care, 577 (17 %) were known to have died, 161 (5 %) had transferred out and 285 (9 %) were classified as LTFU. The mortality rate was 3.22/100 and the rate LTFU was 1.59/100 person years. Table 1 shows the bivariate analysis of factors associated with mortality or LTFU. Male participants represented a higher proportion of those who died or LTFU than those who were retained in care 37 vs 25 %, respectively; (p < 0.001). We also found that WHO stage at treatment initiation (p < 0.001), CD4 cell count at initiation (p < 0.001) hemoglobin values at ART initiation (p < 0.001) and occupation (p = 0.045) were higher in those who died or were LTFU. District of residence at ART initiation was also unequally distributed across the four vital status categories (p < 0.001). However, when expressed as row percentages (rather than column percentages as in Table 1) the proportion retained in care had a fairly narrow range from a low 67 % in Jinja to a high 73 % in surrounding districts (data not shown). Mortality ranged from 14 % in Buikwe to 20 % in Mayuge and LTFU ranged from 4 % in Mayuge to 10 % in “other districts”.

**Table 1:** Cox proportional hazards analysis of factors associated with time to mortality or loss to follow up

List of clinical factors	Univariate		Multivariate	
	HR (95 % CI)	p value	HR (95 % CI)	p value
Age at ART initiation	0.99 (0.98, 1.00)	0.065	1.00 (0.99, 1.01)	0.720
First ARV regimen				
Nevirapine/zidovudine/lamivudine	1.00 (–)	0.018	1.00 (–)	0.003
Efavirenz/stavudine/lamivudine	0.64 (0.39, 1.03)		0.87 (0.53, 1.44)	
Efavirenz/zidovudine/lamivudine	0.61 (0.42, 0.89)		0.74 (0.51, 1.09)	
Nevirapine/stavudine/lamivudine	0.91 (0.74, 1.14)		1.38 (1.08, 1.76)	
Other ARV regimen	1.21 (0.86, 1.71)		1.41 (1.00, 1.99)	
WHO stage				
Stages 1 and 2	1.00 (–)	<0.001	1.00 (–)	0.002
Stage 3	1.52 (1.24, 1.85)		1.36 (1.1, 1.67)	
Stage 4	1.82 (1.3, 2.56)		1.72 (1.21, 2.43)	
CD4 at ART initiation				
≥200	1.00 (–)	<0.001	1.00 (–)	<0.001
50–199	2.21 (1.75, 2.8)		1.86 (1.46, 2.37)	
<50	5.25 (4.07, 6.78)		4.11 (3.13, 5.4)	
District of residence at ART initiation				
Jinja	1.00 (−)	0.879	1.00 (–)	0.562
Buikwe	0.87 (0.65, 1.15)		0.92 (0.69, 1.22)	
Iganga	0.87 (0.65, 1.15)		0.77 (0.58, 1.03)	
Kamuli	0.92 (0.66, 1.28)		0.92 (0.66, 1.28)	
Mayuge	0.95 (0.7, 1.27)		0.95 (0.7, 1.28)	
Other	0.85 (0.55, 1.31)		0.77 (0.5, 1.19)	
Education level				
None/preprimary/other	1.00 (–)	0.587	1.00 (–)	0.400
Higher education	0.89 (0.5, 1.58)		0.73 (0.39, 1.38)	
Some secondary	0.91 (0.67, 1.25)		0.81 (0.58, 1.12)	
Some primary	1.07 (0.82, 1.39)		0.97 (0.74, 1.28)	
Gender				
Female	1.00 (–)	<0.001	1.00 (–)	<0.001
Male	1.62 (1.34, 1.96)		1.6 (1.3, 1.97)	
Occupation				
Peasant	1.00 (–)	0.136	1.00 (–)	0.065
Casual laborer	1.24 (0.9, 1.72)		1.13 (0.81, 1.58)	
None	1.32 (1.02, 1.71)		1.34 (1.02, 1.74)	
Other	1.15 (0.83, 1.6)		1.13 (0.8, 1.58)	
Paid employee	1.01 (0.7, 1.45)		0.92 (0.61, 1.39)	
Vendor/business person	0.86 (0.63, 1.17)		0.79 (0.57, 1.08)	
Year of ART initiation	1.39 (1.29, 1.51)	<0.001	1.32 (1.2, 1.46)	<0.001

In the multivariate Cox proportional hazards analysis of factors associated with time to death or LTFU as in Table 1, male gender [adjusted hazard ratio (AHR) = 1.56; 95 % CI 1.28–1.9] was significantly associated with LTFU or mortality. CD4 cell count <50 (AHR = 4.09; 95 % CI. 3.13–5.36) or 50–199 cells/μL (AHR = 1.86; 95 % CI 1.46–2.37) at ART initiation and WHO stages 3 (AHR = 1.35; 95 % CI 1.1–1.66) or 4 (AHR = 1.74; 95 % CI 1.23–2.45) were also associated with the outcome. Participants who received Stavudine, Lamivudine and Nevirapine, (AHR = 1.38; 95 % CI 1.08–1.76) or atypical ARV regimens (AHR = 1.41; 95 % CI 1.00–1.99) also had an increased hazard of mortality/LTFU in comparison to those who began therapy with Nevirapine, Zidovudine and Lamivudine. As well, the year of ART initiation (AHR = 1.32 per year; 95 % CI 1.20–1.46) was significantly associated with mortality/LTFU. Notably, residence in any of the areas outside of Jinja district, again was not associated with mortality/LTFU in this analysis (p value = 0.562) in comparison to those who resided in Jinja.

We conducted HIV viral load testing on 870 clients of TASO Jinja who had been on ART for more than 4 years and presented for care between June 2012 and August 2013. Of these, we could link 805 (92.5 %) to the electronic database records. These individuals had been receiving ART for a median of 7.0 (IQR 5.0–8.0) years. The similarities and differences in this sample in comparison to the entire TASO-Jinja cohort are presented in Table 2. Note that this sample included six participants who were identified as having transferred out and two who were considered LTFU. The sample had more female participants (77 vs. 69.2 %; p < 0.001) had higher CD4 cell counts at enrollment (97 vs. 69 %; p < 0.001) (74.7 % female vs 69.4 %; p < 0.001) (Table 3).

**Table 2:** Comparison of participants in viral load sub-study in 2012 -13 with other participants in Jinja Cohort

Variable	Total N	Participants in VL sub-study				p value
		No (N = 2540)		Yes (N = 805)		
		N	(%)	N	(%)	
Clinical outcomes	3345					<0.001
Survived and retained in care		1525	(60)	797	(99)	
Dead		577	(23)	0	(0)	
Lost to follow-up		283	(11)	2	(0)	
Transferred out		155	(6)	6	(1)	
Gender	3345					<0.001
Female		1757	(69)	623	(77)	
Male		783	(31)	182	(23)	
CD4 at ART initiation	3099					<0.001
<50		429	(18)	55	(7)	
50–199		958	(42)	278	(35)	
≥200		915	(40)	464	(58)	
Hemoglobin count of participant at ARV initiation or clinical visit	2222					0.025
<8		71	(4)	13	(2)	
≥8		1574	(96)	564	(98)	
WHO stage	2829					0.749
Stage 1		40	(2)	10	(1)	
Stage 2		992	(47)	359	(49)	
Stage 3		913	(44)	318	(43)	
Stage 4		147	(7)	50	(7)	
Occupation	2920					0.384
Casual labourer		202	(9)	59	(9)	
Paid employee		187	(8)	48	(7)	
Peasant		940	(41)	268	(41)	
Vendor/business person		321	(14)	98	15)	
Other		246	(11)	61	(9)	
None		401	(17)	89	(14)	
Education level	2920					0.092
None/preprimary/other		382	(17)	100	(16)	
Some primary		1294	(56)	324	(52)	
Some secondary		545	(24)	174	(28)	
Higher education		75	(3)	26	(4)	
First ARV regimen	3076					0.003
Efavirenz/Zidovudine/Lamivudine		233	(10)	90	(11)	
Efavirenz/Stavudine/Lamivudine		136	(10)	64	(8)	
Nevirapine/Stavudine/Lamivudine		688	(30)	262	(33)	
Nevirapine/Zidovudine/Lamivudine		1034	(45)	327	(42)	
Other ARV regimen		198	(7)	44	(6)	
Year of ART initiation	3345	2006	(2006–2008)	2006	(2005–2007)	<0.001

N = 3345 Jinja cohort participants

**Table 3:** Bivariate analysis of 870 participants with and without viral load <1000 copies/ml

Variable	Total N	Viral load result (copies/mL)				p value
		<1000 (N = 809)		≥1000 (N = 61)		
		N	(%)	N	(%)	
Gender	870					0.083
Female		623	(77)	41	(67)	
Male		186	(23)	20	(33)	
Most important source of income	870					0.399
Agricultural farming		254	(31)	16	(26)	
Wage or salaried employment		93	(11)	8	(13)	
Crafts and trade work		54	(7)	2	(3)	
Petty trade in kiosks stalls and hawking		198	(24)	22	(36)	
None		77	(10)	5	(8)	
Other		133	(16)	8	(13)	
Education level	869					0.127
No education (including do not know)		173	(21)	8	(13)	
Some/completed primary		357	(44)	25	(41)	
Some/completed high school/higher education		278	(34)	28	(46)	
Current marital status	870					0.186
Single/separated/divorced/widowed		481	(59)	31	(51)	
Legally married/co habiting		328	(41)	30	(49)	
Any evidence of CD4 cell count treatment failure?	867					<0.001
No		781	(97)	42	(69)	
Yes		25	(3)	19	(31)	
Current ARV regimen (first regimen)	870					0.084
Nevirapine		626	(77)	53	(87)	
Efavirenz		183	(23)	8	(13)	
Current ARV regimen (second regimen)	870					0.417
3TC/ZDV		744	(92)	54	(89)	
FTC/TDF		1	(0)	0	(0)	
3TC/TDF		63	(8)	7	(11)	
3TC/ABC		1	(0)	0	(0)	
Frequency taking ARV pills	869					0.826
Never		517	(64)	37	(61)	
Once a week		54	(7)	5	(8)	
More than once a week but less than a month		54	(7)	3	(5)	
More than a month		183	(23)	16	(26)	
Adherence (frequency missing ARV pills)	869					0.956
Never or more than a month		700	(87)	53	(87)	
Once a week or more than once a week but less than a month		108	(13)	8	(13)	
Alcohol within previous month	870					0.903
No		739	(91)	56	(92)	
Yes		70	(9)	5	(8)	
Age at time of VL test	870	45	(40–50)	42	(37–45)	0.003
Most recent CD4 cell count (cells/mm^3^)	867	575	(445–757)	253	(118–426)	<0.001
Duration on ART (years)	870	7	(6–8)	7	(5–8)	0.775

N = 870

Of these individuals, 753 (87 %) of the patients had > 90 % adherence to therapy based on self-report of missed doses. A total of 756 (87 %) participants had VLs < 50 copies/mL and 809 (93 %) had VLs <1000 copies/ml. Table 4 shows the bivariate analysis of factors associated with virologic failure, defined as a VL ≥1000 copies/mL. Patients with VLs ≥1000 copies/mL were likely to be older than those with VLs <1000 copies/mL (median 45 Vs 42 years; p < 0.003). A lower proportion of women had virologic failure (67 vs 77 %), but this was only marginally significant (p = 0.083). Those with VLs ≥1000 copies/mL had lower CD4 cell counts at the time of VL testing (median 253 cells/μL) than those with VLs <1000 copies/mL (575 cells/μL; p < 0.001). We found a significant association between virologic failure meeting the CD4 cell count criteria treatment failure as defined by WHO (p < 0.001). Those with virologic failure were also younger than those without (median age 42 vs. 45 years; p < 0.001). There were no significant associations between virologic failure with regimen type, education level, or occupation in the bivariate analysis. Logistic regression analysis revealed that the most recent CD4 cell count [adjusted odds ratio (AOR) = 0.51 per 100 cells/μl; 95 % CI 0.43–0.60] and age (AOR = 0.96 per year; 95 % CI 0.92–0.99) were significantly associated with virologic failure as shown in Table 4. We also found that participants whose initial ART regimen was Nevirapine/Stavudine/Lamivudine had an increased odds of virologic failure (AOR = 1.38; 95 % CI 1.08–1.76) in comparison to those who started with Nevirapine/Zidovudine/Lamivudine (reference category).

**Table 4:** Univariate and multivariate logistic regression analysis of factors associated with virologic failure

	Univariate		Multivariate	
	OR (95 % CI)	p value	OR (95 % CI)	p value
Gender		0.081		
Female	1.00 (–)			
Male	1.65 (0.94, 2.89)			
Education level		0.101		0.132
No education (including do not know)	1.00 (–)		1.00 (–)	
Some/completed Primary	1.47 (0.65, 3.34)		1.51 (0.61, 3.72)	
Some/completed high school/higher education	2.25 (1, 5.04)		2.34 (0.95, 5.76)	
Current marital status		0.265		
Single/separated/divorced/widowed	1.00 (–)			
Legally married/co habiting	1.35 (0.8, 2.28)			
Current ARV regimen (first regimen)		0.091		
Nevirapine	1.00 (–)			
Efavirenz	0.52 (0.24, 1.11)			
Age (in years)	0.95 (0.91, 0.98)	0.003	0.96 (0.92, 0.99)	0.020
Number of child dependents	0.95 (0.88, 1.02)	0.174		
Most recent CD4 cell count (per 100 cells/μl)	0.50 (0.42, 0.59)	<0.001	0.51 (0.43, 0.6)	<0.001

## Discussion

This study of over 3000 participants in the TASO ART Program in Jinja, Uganda demonstrated that more than 69 % of patients who initiated ART from 2004 to 2009 were retained in care after more than 5 years of treatment. This finding demonstrates that high retention rates are possible even in rural, resource-limited settings, particularly when programs are designed to be client-centered. Not only was retention in care quite high only 9 % of participants became LTFU and mortality was relatively low at 17 % and among those retained in care 93 %, had a VL result of <1000 copies/mL, even without access to VL testing for ART monitoring. While some programs have reported similar rates of retention [3, 17, 21, 23, 25, 26, 27, 28], they most often have been reported with study populations with shorter duration of follow-up (17, 21 22, 23, 25, 26] often in the range of 2 years after ART initiation [1, 16, 20], as opposed to the median of 5 years of follow-up in our study. For example, Assefa et al., in the study of outcomes of the ART services in the 55 health facilities in Ethiopia, reported an average 68 % (95 % CI 51–85) of patients retained in care after 2 years of follow-up [1]. The program at TASO, therefore, has retention figures which are among the better performing programs in the region even over longer follow-up periods.

We also found that, the risk for mortality and LTFU did not differ based on the geographic residence of program participants. This suggests that community-based distribution systems in use at TASO has effectively mitigated the time and cost constraints associated with transportation to ART clinic sites which have been demonstrated as barrier to retaining ART patients in care [3, 7, 31].

Among those retained in care and receiving first-line ART, we found a very low prevalence of virologic failure and very few with detectable VL measurements (i.e. ≥50 copies/mL) even in the absence of VL monitoring. While the new WHO 2013 guidelines for the use of ART in resource-limited settings recommend the use of VL testing, it is important to note that ART programs with only routine CD4 testing and good adherence strategies can achieve excellent outcomes and high rates of virologic suppression, even in the absence of such testing. However, it also appears that even the use of these clinical tools available at TASO have not been completely optimized since, we found 44 individuals (5 %) in our sub-study of virologic failure who met criteria for immunologic failure and whom had not yet been switched to second-line therapy. The fact that only 19 of the 44 were found to have VLs ≥1000 copies/mL, suggests confirming virologic failure amongst individuals who meet clinical or immunologic criteria for failure may be a more useful role for VL testing than screening large numbers of participants who are clinically well.

While our mortality rate was fairly low (3.22 per 100 person-years), in comparison to other studies in Africa reporting 5.1 per 100 person-years [6–9, 14] previous studies have shown that underreporting of mortality due to LTFU can bias the reporting of mortality, [20, 22]. However, as LTFU in our study was also low, this does not appear to be the explanation for this observation in our study. Of course, since most mortality on ART occurs within the first few months of initiation, it is also expected that studies with longer follow-up time will also yield lower mortality rates [15, 22, 31].

The rate of LTFU of 1.59 per 100 person-years in our cohort is comparable to that observed in the South Africa studies [17], but relatively lower as compared to the rate observed in Western Kenya which found, 5.1 per 100 person-years of observation [31]. The careful design and scale up of ART in TASO may have contributed to the observed LTFU. This program has grown in size dramatically, with our cohort increasing enrolment about 3-fold from 2004 to 2013.

These findings could be limited because death was ascertained by patient record review. Consequently, deaths, which occurred at home, might have not been documented in the patient card. This may underestimate the rate of mortality. For this reason we combined LTFU with mortality as our outcome. As the data we used for this analysis was routinely collected and entered by clinicians as part of providing clinical care, it lacks some accuracy and was not complete. This may underestimate or overestimate some outcome of interest. As well some variables were extensively missing and we could not include them in multivariate analyses. As well, there is some possibility of misclassification as we observed six patients in the virologic failure sub-study who were classified as having transferred out of the program and two other participants in the sub-study met our criteria of LTFU, despite being in care.

## Conclusion

Among participants enrolled in an ART program for a median of over 5 years, rates of mortality and LTFU were very low and did not differ based on the geographic residence of program participants. This demonstrates that community-based distribution systems can effectively mitigate the time and cost constraints associated with transportation to ART clinic sites. As well, ART programs which only routine CD4 testing and good adherence strategies can expect good clinical and virologic outcomes.

## Methods

### Study design

We conducted a retrospective cohort analysis of all patients >18 years old who initiated ART at the TASO service clinic in Jinja. Jinja clinic is one of the eleven TASO ART clinics in Uganda which serves clients within a radius of 75 km. Between January 2004 and July 2009 the CD4 count cut off point for ART eligibility increased from 200 to 250 cell/ml. The AIDS Support Organization (TASO) has the largest non-governmental ART program in the country with more than 80,000 individuals accessing care and over 65,000 individuals receiving ART through 11 service centers, the ART program has been designed to accommodate the needs of clients since its inception. This has been operationalized in terms of providing clients with much more than just clinic visits and medications. Several types of program delivery models have been implemented and revised over the past 10 years at TASO, including home-based care [11, 30] the use of satellite clinics, community drug distribution [29], as well as more conventional clinic-based approaches. In TASO, patients who are initiated on ART after 6 months of ART treatment are evaluated by clinicians and counselors for down referred to Community Drug Distribution Point (CDDP). A CDDP is a small group of 20–50 patients who have been on ART for at least 6 months, are stable clinically, have disclosed their sero-status to a friend or relative and have consented to down referral to the community for continued ART refill, monitoring by lay workers. The patients themselves identify a place within their community where they converge to receive their refills, ART reviews and evaluation by TASO counselors (lay workers) and six monthly reviews by TASO clinical staff. The group elects a leader who is an expert client who then mobilizes and follows up all the patients in his/her group for adherence and other counseling issues, he is also responsible for up referral of the patients to the facility in case of any illness or suspected medication side effect. In the CDDP, Drug refills are conducted every 2 to 3 months, with clinical assessments conducted at the time of drug refills by health professionals and CD4 cell counts performed every 6 months by laboratory technician. No routine VL monitoring is performed as part of this program. For community-based drug distribution, ART is provided at the community by trained lay workers, who are mostly social workers and teachers supervised by Clinical Services Supervisor.

### Data collection

In June of 2013, we used the clinical database of TASO Jinja, to extract clinical data at enrollment and subsequent visits, the data collected included WHO stage, baseline CD4, CD4 at subsequent visits, HB, distance to the service centre, ART start date and regimen, as well as basic socio-demographic information. The database also records information on known deaths and patients who have transferred out of the program. When necessary, patient charts in use at TASO Jinja were used to supplement the information found in the clinical database. Individual consent was not obtained from the study participants but the patient records were anonymized and de-identified prior to analysis. Additionally, between August 2012 and May 2013, we obtained funding to conduct viral load (VL) tests for clients who attended the clinic or outreach site and who had been on ART for at least 4 years, while still on a first-line ART regimen. The Participants in this component of the study did provide individual written informed consent. The study protocol and consent forms were approved by the Research Ethics Board of the University of British Columbia, the TASO Research Ethics Committee, the Uganda Virus Research Institute and the Uganda National Council for Science and Technology.

### Data analysis

Retention in care was defined by a record of at least one clinic visit in the 6 months before June 1, 2013. We included factors that determined retention and mortality among adults enrolled in HIV service delivery models and conducted bivariate analyse using Kruskal–Wallis and Chi square tests using STATA version 16 to compare clients who remained active program clients throughout the study period, with those who are known to have died or who were LTFU. We used Cox Proportional Hazards Modeling to determine factors associated with the combined outcome of mortality and LTFU, given that the bivariate analysis suggested more similarities between participants in these categories than with those retained in care or transferred out. District of residence was our primary explanatory variables of interest, but we examined other important co-factors, such as CD4 counts at treatment initiation, gender, ethnicity, and other variables for potential inclusion in explanatory models. For the analysis of virologic failure, we calculated the proportion of individuals with virologic failure defined as ≥1000 copies/mL. We then conducted a bivariate analysis with participants classified on the basis of presence or absence of viral failure (defined as a VL of ≥1000 copies/mL) and examined factors independently associated with virologic failure using multivariate logistic regression.

## Abbreviations

TASO: the AIDS Support Organization
CDDP: community drug distribution point
LTFU: lost to follow up
